# The use of *Callitriche cophocarpa* Sendtn. for the reclamation of Cr-contaminated freshwater habitat: benefits and limitations

**DOI:** 10.1007/s11356-020-08887-x

**Published:** 2020-04-29

**Authors:** Joanna Augustynowicz, Ewa Sitek, Tomasz Bryniarski, Agnieszka Baran, Beata Ostachowicz, Małgorzata Urbańska-Stopa, Marek Szklarczyk

**Affiliations:** 1 Department of Botany, Physiology and Plant Protection, Faculty of Biotechnology and Horticulture, University of Agriculture in Kraków, al. 29 Listopada 54, 31-425 Kraków, Poland; 2 Foundation of Research and Science Development, ul. Rydygiera 8, 01-793 Warszawa, Poland; 3 Department of Agricultural and Environmental Chemistry, Faculty of Agriculture and Economics, University of Agriculture in Kraków, Al. Mickiewicza 21, 31-120 Kraków, Poland; 4 Department of Medical Physics and Biophysics, Faculty of Physics and Applied Computer Science, AGH University of Science and Technology, al. Mickiewicza 30, 30-059 Kraków, Poland; 5 Department of Plant Biology and Biotechnology, Faculty of Biotechnology and Horticulture, University of Agriculture in Kraków, al. 29 Listopada 54, 31-425 Kraków, Poland

**Keywords:** *Callitriche cophocarpa*, Chromium, Phytoremediation, Sediments, Tannery effluents, TXRF

## Abstract

This work is the first attempt to evaluate suitability of *Callitriche cophocarpa* Sendtn. (water-starwort) to remove Cr under real-world conditions. Our earlier laboratory-scale studies demonstrated outstanding hyperaccumulation properties of this aquatic higher plant (macrophyte) toward chromium in solution. We introduced *C. cophocarpa* plants into the watershed with sediments heavily polluted (on average 1400 mg/kg d.w. of Cr) by a tannery. The plants grew vigorously and exhibited no physiological or anatomical disorders. Based on chemical fractionations of bottom sediments, we found low Cr bioavailability. The element was strongly associated with the sediments and could be classified into the following fractions (%): oxidizable III (68.2) > residual IV (28.8) > reducible II (1.6) > exchangeable I (1.4). Despite this, Cr content in plant organs at the contaminated sites was 33 up to 83 times greater than in the control leaf/stem and roots, respectively. Altering redox potential during, i.e., sediment deposition on land may change chemical forms of bound metals in a solid phase, and thus further increase Cr phytoextraction by plants. With this in mind, we concluded that the species, being an outstanding Cr accumulator under laboratory conditions, can be useful in the reclamation of Cr-polluted sediments under controlled, oxidizing conditions.

## Introduction

Chromium is widely applied, e.g., in the metallurgical industry in the production of alloys, plating other metals (galvanization), and in tanning. Cr salts exhibit attractive colors (violet, green, yellow, and orange) and are used in manufacturing various types of pigments. Significant contamination with Cr compounds occurs not only in developed countries (e.g., the USA) but also in developing ones (e.g., India) (Shanker and Venkateswarlu 2011). Chromium is present naturally in two stable forms—Cr(III) and Cr(VI). In solutions, Cr(VI) exists as chromate (HCrO_4_^−^) or dichromate (Cr_2_O_7_^2−^) anion. It is a very toxic and extremely strong oxidant, highly soluble in a wide range of pH and thus easily bioavailable. Its harmful effects in cells are due to its oxidizing properties and involve breaking of unsaturated bonds in fatty acids, nucleic acids, and proteins. Contrary to Cr(VI), trivalent Cr at pH 4–10 occurs mainly as cations in the form of insoluble, hydrated hydroxides adsorbable by different organic ligands like humic acids in soils or sediments. Although most cells are impermeable to Cr(III) in non-acidic environment, Cr(VI) is easily reduced to Cr(III) once inside the cell. Cr(III) is an important microelement for mammals, indispensable in maintaining blood sugar level and lipid metabolism (Kotaś and Stasicka 2000). However, apart from its positive effects in mammalian tissues, Cr(III) displays detrimental activity manifested by cross-linking of proteins and DNA (Codd et al. 2001). The equilibrium between Cr speciations depends on pH, concentration of reducers (Fe(II), organic compounds), oxidizing factors (Mn oxides and oxygen), and complexing agents (organic matter) (Kotaś and Stasicka 2000). As concentrations of Cr(III) often exceed permissible levels, the US Environmental Protection Agency (US EPA) recognizes both Cr(VI) and Cr(III) compounds as priority toxic pollutants. Maximum permitted levels of Cr in Poland are 50 μg/L for total Cr in drinking and surface waters and 0.5–1.0 mg/L for sewage introduced into water or ground, respectively. Geochemical (Bojakowska 2001) and ecotoxicological (MacDonald et al. 2000) guidelines are used to assess chromium concentrations in sediments. According to Bojakowska (2001), bottom sediment samples are classified into class I (not polluted), if concentration of Cr < 50 mg/kg; class II (moderately polluted), if concentration of Cr < 100 mg/kg; class III (polluted), if concentration of Cr < 400 mg/kg, and class IV (highly polluted), if concentration of Cr > 400 mg/kg. The ecotoxicological assessment of bottom sediments contaminated with chromium is based on threshold effect concentration (TEC) and probable effect concentration (PEC) methods (MacDonald et al. 2000). The TEC and PEC are applied to evaluate potential ecological risks posed by trace elements (Baran and Tarnawski 2015; Gao et al. 2018). The sediments are considered non-toxic to benthic organisms if Cr concentration is below 43 mg/kg (TEC). The sediments with Cr concentration exceeding 110 mg/kg (PEC) are deemed toxic to benthic organisms (MacDonald et al. 2000). In Poland, the highest level of chromium pollution occurs in the southern regions (Podhale, Western Carpathians), where it has been used for centuries in numerous tanneries (Ślusarczyk et al. 2010). Leather tanning utilizes trivalent Cr salts, especially chromium alum KCr(SO_4_)_2_ and sulfate Cr_2_(SO_4_)_3_∙12H_2_O (Vaiopoulou and Gikas 2012). Due to low concentration limit for chromium it is crucial to eliminate it thoroughly from contaminated effluents. Regrettably, regulations are often violated, and Cr compounds are released into wastewater and groundwater near the tanning facilities. Majority of heavy metal loads supplied to the aquatic environments is accumulated in sediments (Motzer 2005). The sediment load poses a direct threat to benthos. Sediments dissolved due to changes in redox conditions or pH become more bioavailable and thus particularly hazardous for aquatic vegetation (Gao et al. 2018; Singh et al. 2013).

Aquatic plants play a key role in stream ecology. Especially, macrophytes, a heterogeneous group of aquatic plants (on “macroscale”) belong to the factors shaping water depth, chemistry, current velocity, and sediment substrate, thus creating habitats for invertebrates and fish. They also serve as feeding resources (Leuschner and Ellenberg 2017; Riis et al. 2009). Macrophytes have been tested in urban stream and eutrophic river rehabilitation schemes in New Zealand (Larned et al. 2006; Suren 2009), Australia (Paice et al. 2016), and Denmark (Riis et al. 2009). They play a fundamental role in collecting trace elements in aquatic environments, so they are also widely used in phytoremediation. This biological cleaning-up technology is an eco-friendly, low-cost alternative for reclamation of degraded environments. The challenge of trace element bioremediation consists in the fact that, contrary to organic pollutants, they cannot be degraded and have to be removed (extracted). A high number of submerged, emergent, or floating aquatic plants showed some potential to accumulate heavy metallic elements (Dhir 2013). For example, *Eichhornia crassipes* (Mart.) Solms, *Nymphaea* sp., *Lemna* sp., *Salvinia* sp., and *Leersia hexandra* Sw. are known as efficient Cr phytoextractors (Choo et al. 2006; Rezania et al. 2016; Zhang et al. 2007). Submerged macrophytes, like *Callitriche cophocarpa* Sendtn. used in this study, can accumulate more hazardous elements than floating or emergent ones. This is due to greater contact area with the surrounding environment and very thin cuticle (Rezania et al. 2016).

*Callitriche cophocarpa* (water-starwort) is an aquatic, submerged higher plant widespread mainly in Europe and eastern to northern Asia (Russia). *C. cophocarpa* is an evergreen macrophyte growing in both moving and stagnant waters like rivers, streams, creeks, marshes, bogs, and also tundra and Alpine wetlands up to 1400 m a.s.l. (Lansdown 2013). Thanks to its unique physiological properties, the species is extremely capable of purifying waters from excessive concentrations of chromium compounds under laboratory conditions. *Callitriche* hyperaccumulates Cr in physiological tests up to the level of c.a. 30 mg/g d.w., being also significantly resistant to the abiotic stress caused by the presence of heavy metals (Augustynowicz et al. 2010, 2013). The maximum adsorption capacity of dry *C. cophocarpa* biomass reaches 77.1 mg Cr(III)/g, which is higher than for other biosorbents and even commercially used sorbents, including activated carbon or ion-exchange resins (Kyzioł-Komosińska et al. 2018).

Many valuable basic studies focused on phytoremediation, but most of them are still far away from practice (see review by Vangronsveld et al. 2009). Taking into consideration the results of our basic studies, we decided to investigate for the first time the bioremediation potential of *C. cophocarpa* under natural conditions. For the experimental site, we chose the stream near a tannery. We introduced the plants into the sediments heavily contaminated with Cr. We assumed that if the discharge of Cr-rich effluents appeared systematically, the concentration of Cr in the solution increased, which could favor Cr extraction by plants. We were also aware that Cr can change its oxidation state and thus its bioavailability according to mutual interactions, e.g., with microorganisms (Geng et al. 2019). The specific aims of this work were as follows: (1) to transplant *C. cophocarpa* into degraded and Cr-polluted aquatic environment, (2) to evaluate macrophyte acclimatization and physiological status under these conditions, (3) to investigate Cr phytoextraction potential, and finally (4) to determine practical usefulness of the plant species for reclamation of Cr-contaminated bottom sediments.

## Materials and methods

### Material and study sites

**Fig. 1:**
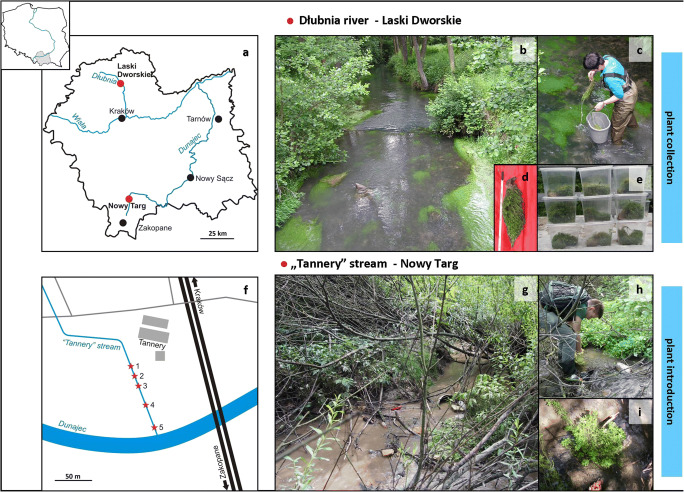
Experimental transplantation of *Callitriche cophocarpa*: (**a**) the map indicating collection (the Dłubia river, Laski Dworskie) and introduction (the “Tannery” stream, Nowy Targ) sites, (**b**) unpolluted Dłubnia river, natural habitat of *Callitriche*, (**c**) collection of plant material, (**d**) a clump, (**e**) plants prepared for transport, (**f**) distribution of experimental sites within the “Tannery” stream (marked with starlets), (**g**) the “Tannery” stream contaminated with chromium, (**h**) plant introduction, and (**i**) a clump after transplantation

The experiment lasted for 4.5 months, from June to October 2016. *Callitriche cophocarpa* was collected from unpolluted Dłubnia river (Laski Dworskie, Miechowska Upland, Nida Trough, Małopolska Upland, southern Poland) (Fig. 1a–c). The river width was 4 m, flow rate 17 m/s, and the depth 25–50 cm. Intensity of light on the river surface measured as quantum flux density (LI-250A, LI-COR Biosciences, USA) was > 100 μmol/m^2^ s. The plant material consisted of similar clumps 30 to 40 cm in length (Fig. 1d), placed separately into transparent plastic containers (Fig. 1e). Then the plants were transported about 130 km to destinations located in Nowy Targ (Orawsko-Nowotarska Basin, Western Carpathians, southern Poland), at an altitude of approx. 580–600 m above sea level (Fig. 1a, f). *Callitriche* was planted in a stream with no geographical name, called “Tannery” for the purpose of this study (Fig. 1g). Significant environmental degradation in the vicinity of this watercourse was due to an adjacent tannery. We selected five different sites along the stream and introduced four clumps at each location (Fig. 1f–i). Each clump (polycormone) consisted of 60–80 structural individuals (rooted shoots); hence, 240–320 plants were introduced at each site, and 1200–1600 in total. The estimated intensity of light before the plant introduction was lower at the sites 1, 2 and 4 (c.a. 40 μmol/m^2^ s^1^), and higher at the sites 3 and 5 (140 μmol/m^2^ s). The light intensity corresponded with canopy cover provided by coastal vegetation (*Fraxinus excelsior* L., *Acer negundo* L., and *Salix* sp.). The width of the “Tannery” bed was c.a. 1.5 m, and its depth was 15–40 cm. The experimental section covered c.a. 100 m. Every 2 weeks we assessed clump survival and clonal growth.

Two independent sets of plant samples were collected: one in the middle and one at the end of the experiment. Since the shoots were intertwined during growth, we could not separate them without mechanical damage. Therefore, mixed plant samples were prepared by harvesting ca.120 rooted shoots from each site.

### Physicochemical analysis of sediment and water

The top layer of the sediment (up to 10 cm) was taken from each experimental site, four sediment samples (100 g each) from every site, then pooled and analyzed in four replicates. Sediment samples were collected both at the beginning and at the end of the experiment. The sediments were digested in a mixture of HCl and HNO_3_ (3:1; v/v) (ultrapure acids, MERCK) in a microwave oven (Multiwave 3000, Anton Paar). Then, optical emission spectrometry (ICP-OES Perkin Elmer Optima 7300 DV) was used for the elemental analysis of the sediment samples. The lower detection limits for elements in the sediment samples were (mg/kg) Cr 0.08; Cd 0.03; Cu 0.12; Pb 0.52; Zn 0.07; and Ni 0.18. BCR-701 (LGC Standards) was used as the reference material for elemental analysis of sediments.

Collection and analysis of water samples took place at the beginning of the experiment, i.e., before the plant introduction. We collected 100 ml of water at each site, pooled the samples, and analyzed in three replicates. Chemical composition of water was analyzed by means of inductively coupled plasma mass spectrometry (ICP-MS) (ELAN 6100, Perkin Elmer) (PN-EN ISO 9963-1:^2001^) and titration methods (PN-ISO 9297:^1994^, PN-EN ISO 17294-1:^2007^). Before measurement of metallic elements with ICP-MS, the water samples were acidified. The spectrometers were calibrated using ICP multi-element standard (Merck). The lower detection limit for elements in the water samples were as follows (mg/l): Na 0.01; K 0.05; Li 0.001; Be 0.0005; Ca 0.05; Mg 0.001; Ba 0.0005; Sr 0.0003; Fe 0.01; Mn 0.003; Ag 0.001; Zn 0.001; Cu 0.001; Ni 0.001; Co 0.0002; Pb 0.0001; Hg 0.0001; Cd 0.0003; Se 0.01; Sb 0.0002; Al 0.005; Cr 0.005; Mo 0.0003; V 0.001; Zr 0.002; Ti 0.02; As 0.001; Tl 0.0001; W 0.0003; Cl 1.0; Br 0.1; J 0.01; SO_4_ 3.0; HCO_3_ 24.4; NO_2_ 0.02; NO_3_ 0.6; PO_4_ 0.002; BO_3_ 0.01; and HBO_2_ 0.01. SPS-SW1 batch 128 or 137 (LGC Standards) was used as the reference material.

### Fractionation of Cr in bottom sediments

The fractional analysis of chromium was performed for sediment samples obtained from experimental location as described above using the BCR three-step method of sequential fractionation proposed by the Commission of the European Communities Bureau of Reference (Baran and Tarnawski 2015; Du Laing et al. 2009). Three fractions of Cr were analyzed: fraction I, exchangeable and acid-soluble form, extractable with CH_3_COOH at 0.11 mol/l, pH = 2; fraction II, forms of Cr associated with free Fe and Mn oxides, extractable with NH_2_OHHCl at 0.5 mol/l, pH = 1.5; and fraction III, Cr forms bound to organic matter, extractable with hot 30% H_2_O_2_ and CH_3_COONH_4_ at 0.5 mol/l, pH = 2. Fraction IV (residue) was calculated from the difference between total content of Cr in the bottom sediments and sum of fractions I–III. Chromium concentration in the solutions was assessed using ICP-OES (ICP-OES Perkin Elmer Optima 7300 DV, USA) method. Total content and sequential extraction of Cr (reached during the recovery) were assessed using BCR-701 (LGC Standards) as a reference material. The percentage of Cr recovery fell between 89 and 98%. The sediment samples were analyzed in four replicates for which relative standard deviations (% RSDs) were below 10%.

### Anatomical studies

Anatomical observations were conducted on leaves, stems, and adventitious roots of plants collected from unpolluted Dłubnia river (control) and the experimental plots at the end of the experiment. Fresh samples were fixed in glutaraldehyde solution (Forsmann 1969) and washed with 0.1-M phosphate buffer. After dehydration in a graded ethanol series, the material was immersed in acetone and embedded in Epon 812 resin (Luft 1961). One micrometer thick sections were cut on Tesla 490A rotary ultramicrotome and stained with 0.1% methylene blue. The specimens were analyzed under Axio Imager M2 microscope (Zeiss, Carl Zeiss, Germany) with Axiovision software.

### Freeze-drying of plant samples

The plant samples used for laboratory analysis (the three paragraphs below) were harvested at the experimental and control (Dłubnia river) sites and immediately transported to the laboratory. Then the material was washed several times with tap water and three times with distilled water. After that the plants were gently dried with filter paper to remove excessive water, immediately frozen at − 80 °C (NU-9334E, Nuaire, Japan), and stored until freeze-drying (maximum for 4 days). Plunged-frozen samples were then transferred to a lyophilizer chamber (Alpha 1–4 Martin Christ Gefriertrocknungsan-lagen GmbH lyophilizer, Germany) and freeze-dried for 72 h at 1.03 mbar and − 20 °C. The lyophilized material was kept at − 20 °C.

### Photosynthetic pigment analysis

Chlorophyll *a*, *b*, and carotenoids were double-extracted from freeze-dried shoots using a modified method described by Świderski (1998). Shoot samples (0.01 g) were homogenized in Eppendorf tubes with 80% (v/v) acetone (POCh, Poland), with a small amount of CaCO_3_ used to neutralize organic acids, and centrifuged for 5 min, at 5400 g and at 4 °C (Rotina 380 R, Hettich Zentrifugen, Germany). The supernatant was collected. In the next step, the sediment (plant tissue) was extracted for the second time using the same procedure. After 15 min of centrifugation at 5400 g and 4 °C, the supernatant was collected again and pooled with the previously collected one up to the total volume of 4 ml. Finally, the absorbance of the extract was measured (UV-Vis spectrophotometer, U-2900, HITACHI, Japan) at 470, 646, and 663 nm. The pigment contents were calculated as recommended by Wellburn (1994).

### Measurements of malondialdehyde (MDA)

Malondialdehyde is a product of cell autooxidation and degradation of unsaturated fatty acids, e.g., in cell membranes (Janero 1990). Its presence is therefore treated as a symptom of stress. MDA can be detected spectrophotometrically due to its ability to form a colorful (pink) adduct with thiobarbituric acid (TBA) at higher temperatures. In our analysis, we used modified protocols described by Zhang and Huang (2013) and Dhindsa et al. (1981). Lyophilized plant shoots (0.01 g) were homogenized in Eppendorf tubes with 1 ml of 0.1% (w/v) trichloroacetic acid (TCA; Sigma-Aldrich, USA) in water and centrifuged for 5 min at 10,000 g and 4 °C (Rotina 380 R, Hettich Zentrifugen, Germany). Then, 0.5 ml of the supernatant was moved to a separate tube and mixed with 4.5 ml of 0.5% thiobarbituric acid (TBA; Sigma-Aldrich, USA) (w/v) in 20% TCA in water (w/v). In the next step, the samples were incubated for 15 min at 95 °C and after that immediately cooled on ice. Finally, the solutions were centrifuged for 10 min, at 10,000 g and 4 °C, and the absorbance of the supernatant was measured (UV-Vis spectrophotometer, U-2900, HITACHI, Japan) at 532 nm. The content of MDA in plant shoots was calculated based on the Beer-Lambert law. The absorption coefficient equaled 155 1/mM cm and molecular weight of MDA is 72.063 g/mol.

### Determination of Cr content in plant organs

Total reflection X-ray fluorescence spectroscopy (TXRF) was used to analyze Cr content in different plant organs (leaves, stems, and roots). Contrary to ICP techniques, which require at least several dozen milligrams for validity, TXRF enables analysis of very small samples (a few milligrams). This technique is useful for multielemental analysis of biological and environmental samples (Turnau et al. 2010), for the elements with atomic number > 19 (potassium to zirconium), and heavy metals. Its detection limit depends on the element. For Cr it was 0.6 mg/kg (dry weight of the biomass). Before the analysis, the plant samples were digested in the pressure digestion bombs (Anton Paar), with the use of nitric acid (suprapure quality). Then, 5 μl of the solution were deposited on an optically flat surface of a reflector and measured after drying. The measurements were run with Nanohunter II (Rigaku) spectrometer, equipped with molybdenum X-ray tube (50 kV, 12 mA). Quantitative analysis was performed after adding selenium as an internal standard. A single measurement time was 2000 s. Citrus leaves, trace elements (LGC Standards), were used as the reference material.

### Statistics

Two sets of experiments were performed as described above and four replicates were conducted for each analysis. Water composition was determined in triplicates at the beginning of the experiment. The results were statistically verified using Statistica 13.1, based on one-way ANOVA and Tukey’s test, at the assumed significance level *α* = 0.05.

## Results

### Qualitative and quantitative analysis of water and sediment

Water analysis preceded transplantation of the macrophytes, and Table 1 presents physicochemical parameters of water at the control and polluted sites. The content of majority of the elements was higher in the “Tannery” watercourse than in the control river. However, according to the standards for surface waters set by the Polish Ministry of the Environment (Journal of Laws 2016), most of the analyzed parameters including chromium did not exceed the limits for class II for both watercourses. This meant good ecological quality of the water at the experimental as well as control sites. High concentrations (> II class) of sodium and chloride corresponded with high electrical conductivity of the “Tannery” watercourse.

**Table 1:** Chemical composition, pH, redox potential (Eh), and conductivity of control and polluted water (“Tannery”). Average value of element concentration are given in (mg/l); *n* = 3 replicates

Element	Control	Tannery
Cations		
Na	2.69	63.29
K	1.48	1.75
Li	0.002	0.003
Be	< 0.0005	< 0.0005
Ca	98.99	72.15
Mg	5.70	9.67
Ba	0.046	0.052
Sr	0.171	0.367
Fe	0.026	0.032
Mn	0.007	< 0.003
Ag	< 0.001	< 0.001
Zn	< 0.002	< 0.002
Cu	< 0.001	< 0.001
Ni	0.0041	0.0046
Co	0.00034	0.00045
Pb	0.00024	0.00033
Hg	< 0.0001	< 0.0001
Cd	< 0.0003	< 0.0003
Se	< 0.010	< 0.010
Sb	< 0.0002	0.0002
Al	0.016	0.013
Cr	< 0.005	< 0.005
Mo	< 0.0003	0.00290
V	< 0.001	0.00120
Zr	< 0.002	< 0.002
Ti	< 0.02	< 0.02
As	< 0.001	< 0.001
Tl	< 0.0001	< 0.0001
W	< 0.002	< 0.002
Anions		
Cl	13.9	112.9
Br	< 0.10	< 0.10
J	< 0.20	< 0.20
SO_4_	16.10	19.90
HCO_3_	282.0	225.0
NO_2_	0.080	0.240
NO_3_	< 0.60	1.70
PO_4_	< 0.002	< 0.002
BO_3_	0.52	0.72
HBO_2_	0.39	0.54
Other parameters		
pH	7.57	7.69
Eh (mV)	321	268
Conductivity (μS/cm)	500	724

The analysis of sediments focused on priority toxic elements, and especially on chromium. The content of Cr and other toxic elements in the sediments of the control river was below limits (Polish Ministry of the Environment, Journal of Laws 2016; data not shown). After 2 months of the experiment (in the middle of August), a rapid heavy rainfall occurred at the experimental sites. This sudden flood caused sediment translocation. Nevertheless, regardless of the analysis date and the site location, the bottom sediments at each “Tannery” site contained high amounts of Cr, i.e., from the average value of c.a. 440 up to almost 4000 mg/kg d.w. (Table 2). Average concentration of other toxic elements (Zn, 156; Cd, 0.58; Cu, 17.8; Ni, 14.5; Pb, 18.7 mg/kg d.w.) allowed for classification of the bottom sediments as not polluted sediments of class I (Bojakowska 2001). In this study, we also assessed potential hazards to living organisms posed by the trace elements in the sediments using threshold effect concentration (TEC; Zn, 121; Cu, 31.6; Pb, 35.8; Cd, 0.99; Ni, 22.7 mg/kg) and probable effect concentration (PEC; Zn, 459; Cu, 149; Pb, 128; Cd, 4.98; Ni, 48.6 mg/kg) values. The PEC was not exceeded in any case, and TEC was above the limit only in the case of zinc. However, Zn concentration was in between TEC and PEC. The sediment samples with element contents between TEC and PEC are potentially nontoxic (MacDonald et al. 2000).

**Table 2:** The average Cr concentration (mg/kg d.w.) in sediments at polluted “Tannery” stands at the beginning (start) and at the end (stop) of the experiment; *n* = 4 replicates

Stand	Start	Stop
1	1521.8 ± 319.0	1332.1 ± 44.8
2	1304.1 ± 505.7	622.1 ± 159.4
3	1018.9 ± 211.1	1447.0 ± 176.6
4	436.7 ± 276.7	798.6 ± 405.1
5	1539.6 ± 217.7	3997.5 ± 828.2

Total chromium concentrations in the sediments at the “Tannery” sites ranged from 440 to 1540 mg/kg d.w. at the start of the experiment and from 662 to 3998 mg/kg d.w. at the end of the experiment (Table 2). According to geochemical quality classes (Bojakowska 2001), the bottom sediments at all experimental sites belonged to class IV, i.e., highly polluted, irrespective of sample collection date. A comparison of Cr concentrations at each location with the sediment ecotoxicology guideline values for TEC (43 mg/kg) and PEC (110 mg/kg) revealed obvious exceedance of PEC values for Cr for all the experimental sediment samples (MacDonald et al. 2000).

The analysis of Cr fractions as well as physiological studies involved samples from the following locations: 1–3, 4, 5, and control (the Dłubnia river). The reason for pooling the samples from sites 1, 2, and 3 was a limited amount of plant biomass required for laboratory analysis due to heavy rainfall that destroyed some clumps (as mentioned above).

Table 3 presents the fractions of Cr bound into the bottom sediments at “Tannery” sites. Chromium was exceptionally strongly bound with the sediments. Average percentage of Cr adsorbed into the sediment and belonging to different fractions was as follows: oxidizable III (68.22) > residual IV (28.76) > reducible II (1.60) > exchangeable I (1.42). Detailed analysis showed predominance of fraction III (organic) at sites 1–3 and 5 and fraction IV at site 4. Chromium of weakly bound fractions II and I was present at very low concentrations of 0.65–2.08% and 0.77–1.08%, respectively, of total Cr content in the sediments.

**Table 3:** Fractions (%) of different binding strength for Cr adsorbed into sediments at polluted “Tannery” stands at the end (stop) of the experiment; *n* = 4 replicates

Stand	1–3	4	5
Fraction I	1.88 ± .0.14	0.71 ± 0.02	1.66 ± 0.19
Fraction II	2.08 ± 0.22	0.65 ± 0.12	2.08 ± 0.31
Fraction III	83.37 ± 3.31	43.27. ± 2.91	78.03 ± 7.83
Fraction IV	12.67 ± 1.22	55.37 ± 1.02	18.23 ± 2.78

Redox potential of polluted sediments at the experimental sites ranged from − 28.15 to − 24.30 mV and pH from 7.15 to 7.22.

### Plant status after introduction

**Fig. 2:**
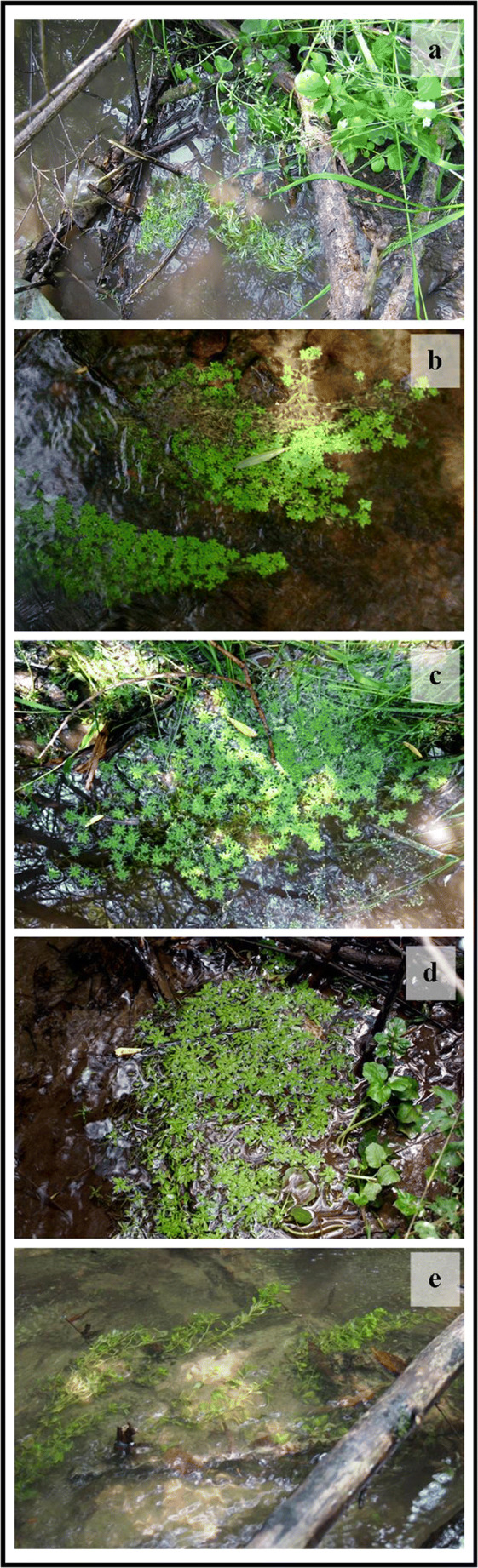
Acclimatization of plants at the polluted sites, representative photographs taken at the site 3 of the “Tannery” stream: (**a**) 9th June 2016, right after the introduction, (**b**) 26th June 2016, (**c**) 10th July 2016, (**d**) 20th July 2016, and (**e**) 29th August 2016, after the heavy rainfall

The plants successfully acclimatized to the experimental sites. Regular monitoring revealed survival of all clumps and their considerable increase in size by c.a. 15–20% every 2 weeks. Finally, after 2 months, all clumps have expanded and formed one integral clump at every site (Fig. 2). Unfortunately, as already mentioned, 2 months after beginning of the experiment a rapid and heavy rainfall occurred. Rise of water level in the stream shifted some sediments and covered several sites destroying some clumps (Fig. 2e). Nevertheless, the plants continued to grow at all experimental sites.

**Fig. 3:**
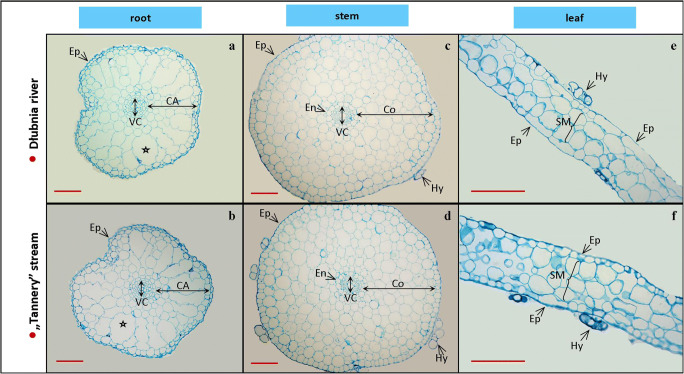
Anatomical structure of *Callitriche cophocarpa* roots (**a, b**), stems (**c, d**) and leaves (**e, f**) collected from the unpolluted Dłubnia river and “Tannery” stream: Ep, epidermis; En, endodermis; CA, cortical aerenchyma (air lacunae marked with starlets); Co, cortex; Hy, hydropote (trichome); SM, spongy mesophyll; and VC, vascular cylinder. Red scale bar = 50 μm

Transplantation and cultivation of *C. cophocarpa* in the “Tannery” stream contaminated with chromium did not cause any changes in plant anatomical structures when compared with control (Fig. 3). Adventitious roots presented primary structure consisting of a single layer epidermis without root hairs, wide cortex with aerenchyma containing numerous lacunae, and a central stele with visible tracheary elements (Fig. 3a). The anatomical structure of stems (Fig. 3b) constituted also of primary tissues: epidermis, extensive cortex, and vascular cylinder (consisting of pericycle, primary xylem, and phloem). The leaves (Fig. 3c) had two-to-three-layer sponge mesophyll with a few small vascular bundles. Multicellular hydropotes were present on lower and upper leaf epidermis, as well as on the stem epidermis. The presence of hydropotes, development of aerenchyma, and reduced amount of vascular tissues are typical for aquatic plants and serve as adaptation to water environment.

**Fig. 4:**
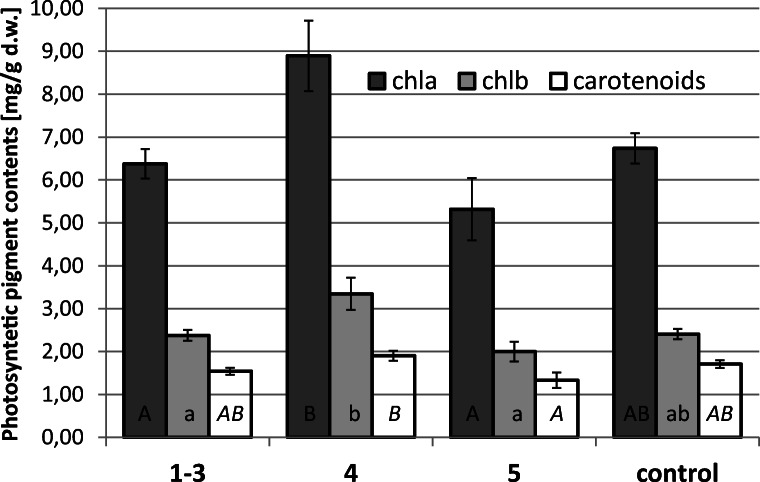
Content (mg/g d.w.) of chlorophyll *a*, chlorophyll *b*, and carotenoids in the control plants (collected from the Dłubnia river) and in the plants from experimental polluted sites (the “Tannery” stream). Different letters denote significant differences (two independent series of experiments were performed with *n* = 4 replicates using ANOVA and Tukey’s test, *α* = 0. 05; *p* = 0.005 chlorophyll *a*; *p* = 0.006 chlorophyll *b; p* = 0.03 carotenoids)

Average concentration of photosynthetic pigments, chlorophyll a (chl *a*), chlorophyll *b* (chl *b*), and carotenoids, as well as the contents chl *a*/*b*, was statistically indistinguishable between plants growing at the polluted sites and the control river (Fig. 4). However, plants growing at site 5 and the control ones exhibited significantly higher carotenoids/(chl *a* + chl *b*) ratio when compared with specimens growing at the remaining sites, i.e., < 0.159; 0.194 > (mean 0.185 ± 0.012) and < 0.140; 0.186 > (mean 0.169 ± 0.014), respectively.

To assess physiological status of plants, we also analyzed the content of malondialdehyde (MDA), which is a stress indicator. We found no significant differences in average content of MDA (mg/g d.w.) in plants from the experimental “Tannery” stream and the control river. However, detailed analysis of MDA concentration in *C. cophocarpa* shoots showed some differences in physiological status between plants from particular sites. Plants grown at “Tannery” 1–3 sites experienced the highest level of cell damage. MDA content in their shoots reached 0.128 ± 0.014 mg/g and exceeded the mean value in the other plants by c.a. 40%. There were no differences in MDA content between plants from the other locations: site 4 (0.093 ± 0.019), site 5 (0.087 ± 0.005), and control (0.090 ± 0.018).

### Cr phytoextraction

**Fig. 5:**
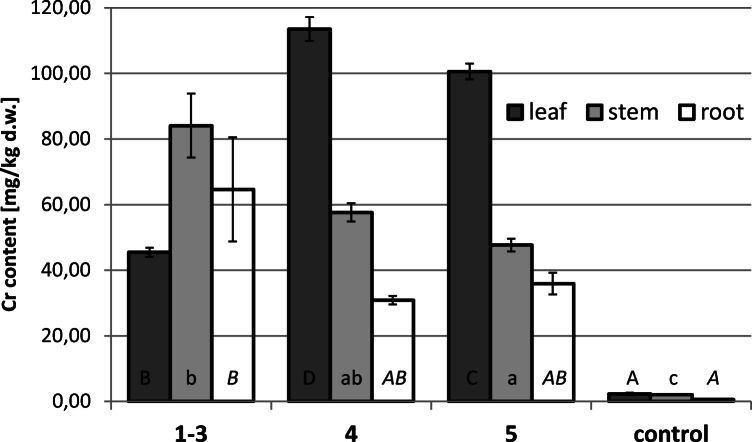
Content of Cr (mg/kg d.w.) in different organs of *Callitriche cophocarpa* collected from the control site (the Dłubnia river) and polluted experimental location (the “Tannery” stream). Different letters denote significant differences (two independent series of experiments were performed with *n* = 4 replicates using ANOVA and Tukey’s test, α = 0. 05; *p* = 0.001 leaf; *p* = 0.0004 stem; *p* = 0.02 root)

Figure 5 shows a detailed analysis of chromium content in individual plant organs. Plants growing at different sites differed in their Cr concentration and distribution. However, regardless of the investigated organ, the plants cultivated at polluted sites accumulated significantly more Cr than controls. Leaves of the plants growing at site 4 accumulated the greatest amount of Cr, the mean value of which was 113.5 mg/kg d.w. Average Cr concentration in the organs of plants cultivated at the experimental sites was as follows (*n* = 16): leaf 76.3 > stem 68.4 > root > 49.0 mg/kg d.w. This was 33 up to 83 times higher than in the control leaf/stem and roots, respectively. Similar pattern of Cr distribution, i.e., leaf > stem > root, was observed in the plants growing at sites 4 and 5 of the “Tannery” and in the control river. Plants growing at the polluted sites 1–3 exhibited different patterns of Cr accumulation. Nevertheless, a bioconcentration factor (BCF) that is defined as the element concentration in plants (mg/kg d.w.) divided by its concentration in the sediment (mg/kg d.w.) was < 1 in all organ samples. BCF values were similar in the plants from the “Tannery” stream (from 0.01 in roots to 0.18 in leaves) and in the control organs (from 0.04 in roots to 0.15 in leaves).

## Discussion

The inspiration for this study came from our earlier laboratory-scale experiments showing outstanding capacity of *Callitriche cophocarpa* to extract chromium from aqueous solutions (Augustynowicz et al. 2010, 2013; Kyzioł-Komosińska et al. 2018). So far, no field study has investigated the use of this widespread aquatic higher plant in phytoremediation of Cr-polluted sites.

The main aim of this study was to determine Cr-bioremediation potential of *C. cophocarpa* under natural conditions. The key factor in successful bioremediation is successful acclimatization confirmed by good physiological status of the plant. Therefore, we had to implement specific targets, before we answered the question if *C. cophocarpa* can be applied in field conditions.

The first specific target was to transplant *C. cophocarpa* into the Cr-polluted sites. Transplanting of macrophytes is considered a cost-effective rehabilitation method improving the ecological status of degraded streams. The experimental sites, located c.a. 130 km from their original location, were chosen for their extremely high Cr content in bottom sediments. Plants were introduced to the sites located next to the tannery, within the stream that flows directly into the Dunajec. The Dunajec serves as one of water sources for Nowy Targ population (c.a. 33,000 citizens). The extremely polluted location of the experiment is not recorded in any research literature or, as far as we know, in any type of documents prepared by local authorities. *C. cophocarpa* was successfully introduced by Riis et al. (2009) into two physically degraded streams in Eastern Jutland, Denmark. However, there are no literature reports on the acclimatization of the *Callitriche* genus in heavy metal polluted environment. Following transplantation, we tested the acclimatization capability of the macrophytes for 5 months. Survival rate of the introduced clumps was 100%, similarly to that reported by Riis et al. (2009). Moreover, the plants grew up vigorously in the Cr-contaminated habitat. Abiotic parameters determining macrophyte occurrence include light, temperature, water nutrient content, substrate characteristics, water movement, and its disturbances like flood (Bornette and Puijalon 2011). Detailed analysis of these parameters was outside the scope of this work but we found that none of them, apart from the flood, limited acclimatization of the plants. In natural habitats, periodic uprooting due to flood events and associated sediment translocation is typical (Sand-Jensen and Madsen 1992) for seasonal population dynamics of this species. The “Tannery” stream enters the Dunajec, a right tributary of the Vistula. As it originates in the nearby Tatra Mountains, it is characterized by fast flow rate and great periodic fluctuations of water levels, especially between May and August (Wyżga et al. 2012). In conclusion, occasional decline in *Callitriche* population is a natural process, even though unfavorable for phytoremediation under environmental conditions.

The next specific target of our study was to compare physiological status of the plants growing under stress with those collected from the original location. Apart from very high concentration of Cr in the sediments, we also noticed high levels of sodium and chloride ions in the “Tannery” watercourse. Permissible levels of heavy metals were not exceeded in the aqueous phase. Higher concentrations of Na and Cl may be a consequence of sewage release from the nearby tannery.

As the first step, we assessed the plant morphology and anatomy. Singh et al. (2013) reviewed negative effects of Cr on shoots and roots at the organ, tissue, and cell structure levels. They reported the presence of necrotic lesions, stunted and brownish roots, destruction of root cortex, shoot structural abnormalities, leaf chlorosis, and changes in mesophyll arrangement in many aquatic and terrestrial plant species. We did not find any disturbances in root and shoot anatomy and morphology when compared with control plants, even though these organs accumulated considerably more Cr. Our earlier 4-week laboratory-scale experiments showed no disturbances in *C. cophocarpa* growth and morphology following exposure of its shoots to 50-μM Cr(VI) (2.6 mg/L) (Augustynowicz et al. 2010). Plants exposed to this concentration of chromium accumulated after four weeks 995 mg/kg d.w. of this element. Symptoms of Cr toxicity, such as shortening of internodes and changes in the leaf shape and surface appeared in plants immersed in 100-μM Cr(VI) (5.2.-mg/L Cr(VI). The lack of anatomical distortions in the present study may be explained by two factors: Cr concentration in water and the function of *Callitriche* roots. So far, *C. cophocarpa* was only exposed to Cr solutions in a laboratory. In this field study, Cr concentration was very low in water (Table 1), but extremely high in the bottom sediments. *Callitriche* roots (Fig. 2a) have a simplified structure, typical for some hydrophytes. Madsen and Cedergreen (2002), who investigated submerged plants, i.e., *Callitriche*, demonstrated that these macrophytes uptake chromium via shoots. Roots of the submerged macrophytes provide only physical support for the plants and their function in mineral uptake is marginal. Indeed, working with the plant shoots in hydroponic cultures, we found that Cr was accumulated by hydropotes (trichomes) present on the leaf and stem epidermis (Augustynowicz et al. 2014). Therefore, it seems that *Callitriche* very efficiently extracts Cr from water but is probably unable of extracting Cr from sediments or/and mobilize it from the sediments to the solution.

Apart from anatomical and morphological features, we also analyzed the content of photosynthetic pigments. A negative influence of heavy metallic compounds on photosynthesis is well documented in the literature. Metal ions negatively affect chloroplast ultrastructure, photosynthetic electron transfer, photosynthetic pigment content, and activity of Calvin cycle enzymes (Piwowarczyk et al. 2018; Tokarz et al. 2018; Singh et al. 2013). These disturbances may be caused, i.e., by direct or indirect oxidative stress triggered by heavy metal uptake and further reactions inside the plant cell. Plants growing at the experimental sites did not show any significant decrease in photosynthetic pigment content when compared with control. However, we noticed a significantly higher carotenoid to total chlorophyll ratio in the plants growing at site 5 and the control ones. This was most likely related to a more efficient xanthophyll cycle. Light intensity was higher at these sites (please compare “Material and study sites” subsection), and the function of the xanthophyll cycle is to dissipate the excess of light energy in order to protect the photosynthetic apparatus (Demmig et al. 1987).

Lipid peroxidation as a consequence of oxidative stress is another toxic physiological effect of chromium (Singh et al. 2013). Oxidation of double bonds in unsaturated fatty acids present in cell membranes may result in malondialdehyde (MDA) formation (Bartosz 2009), what was also found in aquatic plants like *Lemna minor* L. (Varga et al. 2013) and *Eichhornia crassipes* (González et al. 2015). Therefore, the physiological condition of plants in this study was also assessed by measuring MDA content. So far, no laboratory tests have been carried out regarding MDA levels in *C. cophocarpa* plants subjected to abiotic stress. We saw no differences in MDA levels between plants growing at the sites 4 and 5 and the control ones, while plants from sites 1–3 in the “Tannery” stream showed enhanced concentration of MDA (Fig. 5). The reason for higher MDA concentration in the shoots of these plants could be due to their location, as the sites were characterized by considerable amounts of the bottom sediments. The sediments successively covering the shoots caused some mechanical damage to the plants. Hence, the oxidative stress triggering MDA formation may also originate from mechanical injuries of the plants (Gould et al. 2002) growing at this particular location. Considering the described morphological, anatomical, and physiological status of the plants, we can state that *Callitriche* is very resistant to high concentrations of Cr in the sediments. It demonstrated comparable viability at highly contaminated and non-contaminated sites. These findings, obtained for the first time in the field conditions, confirmed our earlier data from laboratory investigations (e.g., Augustynowicz et al. 2010).

The final specific target of this study was to evaluate Cr phytoextraction capacity by *C. cophocarpa* from Cr-contaminated bottom sediments. We did not observe any relationships between the concentration of Cr in the sediments measured at the beginning of the experiment and after its completion regardless of the experimental site (Table 2). An obvious explanation of this were the disturbances in water movement (flood) that caused the sediment translocations. Despite that, the plants were exposed to Cr concentration significantly exceeding toxicity class IV, i.e., 400 mg/kg d.w. (Bojakowska 2001) during the entire experiment. Mean Cr concentration at the experimental sites was 1402 mg/kg d.w., whereas the maximum average accumulation in plants reached 113.5 mg/kg d.w. As per definition, Cr-hyperaccumulator is a plant that grows in a natural environment and accumulates > 300 mg/kg d.w. of the element while exhibiting bioconcentration factor (BCF) > 1 (Van der Ent et al. 2013). By this definition, there are only a few species known as hyperaccumulators of chromium, occurring in high temperature regions: *Dicoma niccolifera* Wild and *Jamesbrittenia fodina* (Wild) O. M. Hilliard from Zimbabwe (Wild 1974), a moss *Aerobryopsis wallichii* (Brid.) M. Fleisch. from New Caledonia (Lee et al. 1977), and a single aquatic (wetland) species *Leersia hexandra* growing in southern China (Zhang et al. 2007). In this study, no organ of *Callitriche* accumulated chromium to the degree higher than the defined one together with achieving BCF > 1. Our laboratory experiments indicated that *Callitriche* was capable of accumulating 995 mg/kg d.w. of Cr(VI) from aqueous solutions, with BCF equal 27, and with no toxicity symptoms (Augustynowicz et al. 2010). When growing in 4-mM Cr(III) solutions, the species accumulated about five times more Cr, up to c.a. 30,000 mg/kg d.w., with BCF up to 517 (Augustynowicz et al. 2013). Therefore, trying to explain these unexpectedly low BCFs and relatively low Cr accumulation under the natural conditions, we performed a chemical fractionation of Cr bound to the sediments.

The chemical fractionation identifies main binding sites and quantifies the strength of a metal binding to particulates. The strength of the metal binding determines mobility of particular elements and their further bioavailability. There are different methods of chemical fractionation. BCR method of sequential fractionation used in this study enabled us to determine four fractions of Cr bound to the sediments: I, exchangeable forms; II, forms of Cr associated with free Fe and Mn oxides; III, forms bound to organic matter; IV, residual phase of metals bound into lithogenic minerals (Baran and Tarnawski 2015). Fraction III is regarded very important for Cr distribution due to high affinity of Cr to organic matter (Baran and Tarnawski 2015; Shaheen and Rinklebe 2014). Due to different ground geochemistry, we found slight differences between particular locations; however, on average, more than 96% of Cr bound to the sediments appeared in fraction III (68.2%) and IV (28.7%) (Tab. 3). It suggests strong association of Cr with the bottom sediments and its highly limited bioavailability. The weakly bound fractions I and II constituted on average 1.4% and 1.6%, respectively. According to the Risk Assessment Code (Singh et al. 2005) we found low risk of chromium release from the bottom sediments.

Organic matter content, pH, redox potential, and the content of inorganic and organic pollutant are the main factors determining phytotoxicity of the sediment (Du Laing et al. 2009). It seems that high concentrations of Cr in the sediments did not threaten *C. cophocarpa* vegetation at the experimental sites. This was mainly due to the sorption of chromium by organic particles of the sediments, which also limited Cr availability to the plants. PEC values for Cr, exceeded at all experimental sites, negatively affected benthic organisms. Strong Cr-sediment binding rate combined with roots not participating in mineral uptake were the reasons of relatively poor Cr accumulation by *Callitriche*. Due to their simplified structure, the plant roots most likely do not have the capacity to efficiently change the redox potential. Moreover, alkaline pH of water and the sediments fosters Cr precipitation (Kyzioł-Komosińska et al. 2018). Based on the physicochemical properties of Cr in low redox potential and alkaline pH of water and sediment as well as strong association of Cr into the sediment, we can predict that Cr(III) was the dominant form at the polluted sites (Kotaś and Stasicka 2000). However, changes in the redox potential, e.g., during sediment deposition on the land, may alter chemical forms of metals bound in the solid phase form. This phenomenon is important when polluted sediments are subjected to oxidation, as it facilitates the element release into the aqueous phase according to the changes of Cr speciation from tri- to hexavalent. As a consequence, the metallic element becomes bioavailable to aquatic plants. Finally, we can speculate that environmental threat caused by chromium released from the sediments would be related to the following factors: (1) flood, due to translocation of the sediments to drinking water reservoirs; (2) pH changes, as acidic pH increases mobility of heavy metallic compounds/ions; and (3) sediment oxidation promoted by sediment deposition and/or land drainage.

To sum up, we can assume that discharges of Cr-rich effluents appear in water; thus, the concentration of Cr in aquatic systems may periodically increase. Moreover, Cr can change its oxidation state and thus mobility due to complex factors described above. Since *Callitriche* is an outstanding Cr phytoaccumulator from the solution (Augustynowicz et al. 2010), occurrences of highly mobile/bioavailable Cr(VI) in water would obviously favor Cr extraction by these plants. Therefore, we believe that presence of this plant species in Cr-polluted freshwater habitat would mitigate the harmful effects of chromium after its mobilization from sediments.

## Conclusions

The study demonstrated a successful acclimatization of *Callitriche cophocarpa* to strongly Cr-polluted environment. The plants grew without any signs of degeneration in their anatomical structures and maintained their physiological status at a similar level as the control plants. The species accumulated Cr in its shoots and roots, but due to poor Cr bioavailability in the sediments and the simplified root structure, bioconcentration factors were low. Extraordinary physiological tolerance to extremely high Cr concentrations in water, as shown in laboratory conditions, and in the bottom sediments, as described in this paper, makes this macrophyte a promising candidate for the reclamation of Cr-contaminated aquatic systems. Flood was the only environmental factor that affected the plant acclimatization. For use in the phytoremediation practice, we suggest to cultivate this species under controlled (pH, redox potential, water disturbances) conditions.
